# Age Determination of *Chrysomya megacephala* Pupae through Reflectance and Machine Learning Analysis

**DOI:** 10.3390/insects15030184

**Published:** 2024-03-10

**Authors:** Xiangyan Zhang, Hongke Qu, Ziqi Zhou, Sile Chen, Fernand Jocelin Ngando, Fengqin Yang, Jiao Xiao, Yadong Guo, Jifeng Cai, Changquan Zhang

**Affiliations:** 1 Department of Forensic Science, School of Basic Medical Sciences, Central South University, Changsha 410013, China216506003@csu.edu.cn (J.X.);; 2 School of Basic Medical Sciences, Central South University, Changsha 410013, China; 3 Xiangya School of Medicine, Central South University, Changsha 410013, China

**Keywords:** *Chrysomya megacephala*, pupa, hyperspectral imaging, machine learning

## Abstract

**Simple Summary:**

The duration of pupa development on a cadaver holds the potential for estimating the time of colonization (TOC), which is often correlated with the postmortem interval (PMI) of decomposed bodies. Establishing an objective, precise, and efficient method for inferring pupa age has become paramount in forensic entomology. In this study, we observed temporal variations in the reflection spectrum of *Chrysomya megacephala* (Diptera: Calliphoridae) pupa, detectable through hyperspectral imaging (HSI). Additionally, we proposed that the eXtreme Gradient Boosting Regression (XGBR) model represents an optimal approach for estimating pupa development time based on HSI data.

**Abstract:**

Estimating the age of pupa during the development time of the blow fly *Chrysomya megacephala* (Diptera: Calliphoridae) is of forensic significance as it assists in determining the time of colonization (TOC), which could help to determine the postmortem interval (PMI). However, establishing an objective, accurate, and efficient method for pupa age inference is still a leading matter of concern among forensic entomologists. In this study, we utilized hyperspectral imaging (HSI) technology to analyze the reflectance changes of pupa development under different temperatures (15 °C, 20 °C, 25 °C, and 30 °C). The spectrograms showed a downtrend under all temperatures. We used PCA to reduce the dimensionality of the spectral data, and then machine learning models (RF, SVR-RBF, SVR-POLY, XGBR, and Lasso) were built. RF, SVR with RBF kernel, and XGBR could show promise in accurate developmental time estimation using accumulated degree days. Among these, the XGBR model consistently exhibited the most minor errors, ranging between 3.9156 and 7.3951 (MAE). This study has identified the value of further refinement of HSI in forensic applications involving entomological specimens, and identified the considerable potential of HSI in forensic practice.

## 1. Introduction

Accurately deducing the developmental time of necrophagous insects is a critical prerequisite for predicting time of colonization (TOC), which could help estimate postmortem interval (PMI) [1,2,3]. For cadavers with a PMI of less than 24 h, observations of early postmortem phenomena, analysis of body chemical composition, and the application of forensic entomology methods can all determine the PMI [4,5,6]. However, when the time since death surpasses 72 h, the reliability of traditional indicators is compromised due to stabilized postmortem and chemical changes in the body. In such instances, insect evidence emerges as the sole dependable indicator [7]. Common methods for inferring insect developmental time include measuring larval body length, identifying developmental stages (larvae, pupa, adult), and calculating cumulative developmental degree days [8,9,10,11,12,13]. Nevertheless, these indicators are primarily suited for larval developmental time inference. The transition of necrophagous fly larvae into the pupa stage poses challenges in observing morphological temporal changes, making it difficult to determine their developmental time [14,15]. Therefore, there is a need to explore more suitable methods for inferring the pupa stage.

The pupa stage, a pivotal period in the metamorphosis of holometabolous insects, bridges the gap between the larval and adult stages. It also represents the most common form of certain fly species at crime scenes, constituting nearly 50% of the non-adult stage found [16]. Forensic entomologists currently employ various methods to infer pupa age, including gene expression analysis [17] and morphological examination of external and internal developmental changes using conventional light microscopy [18], scanning electron microscopy [19], micro-computed tomography [20], gas chromatography-mass spectrometry (GC-MS) [21], and optical coherence tomography [22]. However, these methods present challenges, such as the need for expensive analytical equipment, insufficient baseline reference data, and, notably, many of these methods involve destructive analysis of fly pupa during examination, compromising the integrity of pupa collected at the crime scene as evidentiary material and hindering reevaluation in judicial practice. Establishing an objective, accurate, and efficient method for pupa age inference has become a leading concern among forensic entomologists.

Hyperspectral imaging (HSI) is a sophisticated technology that captures and analyzes fine spectra details at each point in a spatial area [23]. It can detect unique spectral features of individual objects at different spatial positions which are otherwise visually indistinguishable [24]. The technical characteristics of HSI include high spectral resolution, continuous, narrow-band image data, and the construction of a three-dimensional (3D) hyperspectral cube containing two-dimensional spatial information (x rows and y columns) and one-dimensional spectral information (wavelength). This cube reflects the spectral data generated by the physical structure and biochemical composition differences in the sample, revealing structural and physiological information [24]. HSI is characterized by its correspondence between spatial and spectral information, non-invasiveness, and high throughput, and it is widely used in agriculture production monitoring, food safety, medical examinations, and other fields [25,26]. HSI is a non-destructive and non-invasive method that detects and times stress responses in adult beetles [27]. *Calliphora dubia* and *Chrysomya rufifacies* could be distinguished by using a linear discriminant classification model based on HSI data, and the accuracy rate could reach up to 92.5% [28].

Our previous research found that the biochemical profile of the insect cuticle and puparium could change over time, as detected by FTIR and GC-MS [21,29]. There is potential for discernible alterations in the reflectance patterns of the puparium as time progresses, which could serve as indicators for estimating the pupa age. The composition of the insect (puparium and larvae) cuticle is highly dynamic according to gene regulation [30]. The intricate functions of the insect cuticle underscore the notion that its composition is intricately linked to internal physiological processes [31]. Detectable alterations in the biochemical makeup of the cuticle during blow fly pupariation contribute to species differentiation and play a role in age determination. Consequently, we anticipate that distinct differences in reflectance profiles, discernible through HSI, will manifest in pupa at varying ages and temperatures.

HSI technology may have several potential advantages compared to existing methods for pupa age inference. Firstly, HSI possesses non-invasive analytical capabilities, applicable to live specimens and unique specimens. Secondly, HSI is more economical and convenient than techniques like differential gene expression detection and GC-MS; the HSI camera is compact, portable, operationally simple, and does not require specific reagents nor pretreatment during detection. It can capture images at the crime scene and potentially achieve automated classification. Finally, HSI technology data are based on sample reflectance, providing an objective basis for discrimination. This feature opens the possibility of developing image analysis support software, reducing the professional knowledge required for interpretation. Thus, hyperspectral imaging technology holds ample theoretical basis and unique technical advantages for pupa age inference in necrophagous fly species, providing a potential solution to the drawbacks of existing methods and establishing a more efficient and convenient inference approach.

In this study, we utilized a laboratory population of the blow fly *Chrysomya megacephala* (Fabricius, 1794) (Diptera: Calliphoridae), collecting pupa samples under constant temperature conditions of 15 °C, 20 °C, 25 °C, and 30 °C. We employed HSI technology to collect hyperspectral data and then Principal Component Analysis (PCA) for dimensionality reduction. After that, machine learning models were used to predict the development time of *Ch. megacephala* pupa.

## 2. Materials and Methods

### 2.1. Sample Collection

Adult specimens of *Ch. megacephala* were collected in Changsha (28°12′ N; 112°58′ E), Hunan, China, in September 2021 and conserved in Guo’s laboratory. The species were identified according to mitochondrial DNA (OR739421 and OR739422) [32]. The adults were reared in a nylon box (35 × 35 × 35 cm^3^) and assigned to an artificial climate cage (250A GPL, Shen Zhen Ren Gong. Ltd., Tianjin, China) with the temperature set at 25.0 ± 1.0 °C, relative humidity 70%, and a 12:12 h Light/Dark photoperiod cycle. Water was provided at all times through soaked cotton, and they were fed a 1:1 mixture of milk powder (Guilin Zhiren Food Industry Co., Ltd., Guilin, China) and sugar (white granulated sugar) in Petri dishes (9.0 × 1.5 cm^3^ diameter). 

Pork lung (50 g) was used to stimulate oviposition. After two hours, egg masses were collected and carefully divided into four parts. Each portion was reared in a plastic bowl (18 cm diameter, 5 cm height) with a relative quantity of fresh pork lung. These plastic bowls were then moved into fly-rearing cages (35 × 35 × 35 cm^3^) covered with 2 cm of sand until pupation. The fly-rearing boxes were placed into the artificial climate incubator (LRH-250-GSI, Taihong Co., Ltd., Shaoguan, China) at constant temperatures of 15 °C, 20 °C, 25 °C, and 30 °C, with 70% RH and a photoperiod 12:12 L/D cycle. Fresh pieces of pork lung were replenished 1–3 times a day according to the consumption by the larvae. When the larvae developed into the pupal stage, which could be distinguished based on the color of the puparium, the samples were collected every day until eclosion under each temperature. The pupa was immersed in hot water at 90 °C for 30 s and then preserved in 75% ethanol under 4 °C.

### 2.2. Hyperspectral Imaging

The HSI was collected with a 15 W, 220 V LED artificial light bulb under controlled laboratory conditions. These bulbs were installed in a room with a temperature of 24 ± 2 °C and a relative humidity of 30–40%. The pupa collected at different temperatures were placed on a custom-designed scanning platform. The platform was connected to a hyperspectral imaging device, and the instrument was controlled using the SOC710-VP data acquisition software (V6.0.3). The HSI was acquired and displayed as a hyperspectral image cube.

### 2.3. Hyperspectral Image Reflectance Transformation

For the acquired hyperspectral image cube, the SRAnal710e (V3.5) software provided with the instrument was utilized for reflectance transformation to obtain relative reflectance. Relative reflectance is typically the ratio of reflectance obtained from a white calibration board (white = 1) to that obtained from complete darkness (dark = 0). In this experiment, we selected a calibration board with a grayscale of 18% for additional calibration, ensuring consistency in data acquisition over a period. Specifically, the calibration board was scanned before and after each operation to obtain its average reflectance curve. This procedure aimed to ensure that changes in the pupa, observed due to environmental and human factors, were less than 3% during each observation, thereby confirming that changes in pupa reflectance were the key factor in presenting experimental results. The HSI images and post-reflectance transformation were opened using ENVI5.3 software. The region reflectance extraction feature was employed to select the central region of each fly pupa, and output processing was executed to obtain the average relative reflectance for each pupa.

### 2.4. Data Analysis

The initial dataset underwent dimensionality reduction through PCA, followed by training and validation using machine learning algorithms. PCA is an orthogonal linear transformation that eliminates correlated variables, producing a new set of uncorrelated variables known as principal components (PCs). Consequently, the transformed dataset exhibits a reduced number of PCs compared to the original set. The weights used for converting variables into PCs, referred to as “loadings”, signify the variables contributing the most to the transformation. The decision on the number of PCs utilized in the transformation was guided by achieving a cumulative interpretation rate exceeding 99%. 

For regression models, various machine learning models, including Random Forest (RF), Support Vector Regression with Radial Basis Function kernel (SVR-RBF), Support Vector Regression with Polynomial kernel (SVR-POLY), eXtreme Gradient Boosting Regression (XGBR), and Least Absolute Shrinkage and Selection Operator (Lasso), were chosen. In the experimental setup, the dataset was divided into a training set (80%) and a testing set (20%), where the former facilitated model training and the latter assessed the model’s performance and generalization ability. The selected algorithms learned the mapping relationship between input and output during training. The GridSearchCV algorithm optimized the model and identified optimal hyperparameters, and the model underwent scoring through 7-fold Cross-Validation. Four metrics, namely Mean Square Error (MSE), Root Mean Square Error (RMSE), Mean Absolute Error (MAE), and R-Square Score (R2 score), were chosen to evaluate the model. MSE and RMSE, while sensitive to outliers, provide insights into the distribution of prediction errors, with RMSE offering values closer to the predicted outcome. MAE, less sensitive to outliers, cannot reflect the distribution of prediction errors. The selection of the most suitable algorithm was based on the comprehensive analysis outlined above, all implemented using Python 3.

## 3. Results

### 3.1. Hyperspectral Imaging Spectroscopy of Ch. megacephala Pupa Stage under Different Temperatures

**Figure 1:**
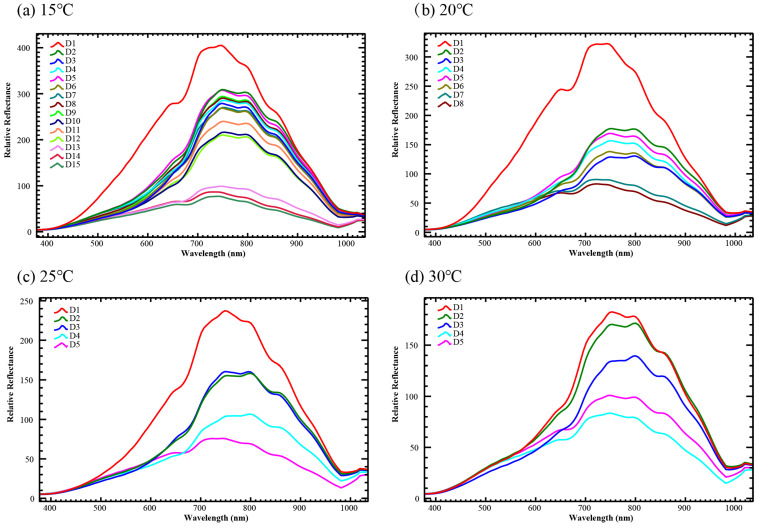
The relative reflectance changes with pupa development under different temperatures. (**a**) The pupa reared under 15 °C; (**b**) the pupa reared under 20 °C; (**c**) the pupa reared under 25 °C; (**d**) the pupa reared under 30 °C. Different colors line means different pupa development day.

In this study, the pupa samples of *Ch. megacephala* were collected at temperatures of 15 °C, 20 °C, 25 °C, and 30 °C. After processing using ENVI 5.3 software, the average reflectance spectra were obtained for each pupa in the wavelength range of 377–1035 nm, with 128 bands. Spectrograms were generated to visualize the daily average reflectance data for *Ch. megacephala* at each temperature (Figure 1). In the 15 °C group, the average reflectance on the first day was the highest compared to other groups, followed by days 2–12, showing a complex arrangement of reflectance. Finally, days 13–15 exhibited a gradual decrease in spectral reflectance. A similar pattern was observed in the 20 °C and 25 °C groups, while at 30 °C, the average reflectance gradually decreased with pupa development, with a slight increase on the last day.

In summary, the spectrogram indicates that irrespective of temperature, the average reflectance is highest on the first day of pupation, and is significantly higher than other periods. Lower temperatures show more significant differences in average reflectance between early and late pupa stages. Over time, the average reflectance at each temperature exhibits a decreasing trend.

### 3.2. Machine Learning Models for Estimating the Development Time of Pupa

In this section, the dataset underwent preprocessing using the T^2^ Hotteling test on the original dataset to identify significant deviations from the central position of given observation values [33]. Outliers (at a 99% confidence level) were removed, and the remaining samples were used for subsequent machine learning analysis, as detailed in Table 1. Based on temperature, the data were divided into 15 °C, 20 °C, 25 °C, 30 °C, and ALL (including all temperatures) groups. The analysis utilized the accumulated degree days (ADD) as the regression target, and regression models were constructed between spectral data and ADD. ADD could be used to compare the development time under different temperatures. After preliminary experiments, a temperature of 10.43 °C was selected as the absolute developmental zero (D0).

**Figure 2:**
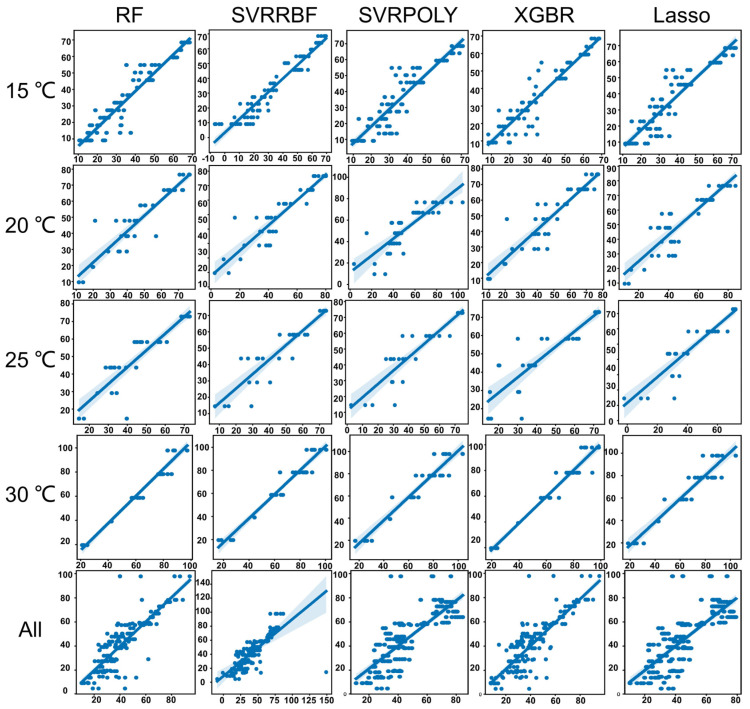
The prediction results (testing set) of reflectance-based age determination with different machine learning models under each group (15 °C, 20 °C, 25 °C, 30 °C, and all temperatures group). The vertical axis of each graph is the actual value, and the horizontal axis is the predicted value, both of which are ADD. RF: Random Forest; SVRRBF: Support Vector Regression with Radial Basis Function kernel; SVRPOLY: Support Vector Regression with Polynomial kernel; XGBR: eXtreme Gradient Boosting Regression; Lasso: Least Absolute Shrinkage and Selection Operator.

Following data preprocessing, PCA was applied for dimensionality reduction. The number of principal components (PCs) used in the model were selected according to the cumulative explanatory rate > 99%, which means the data features were reduced from the original 128 to 6 (15 °C), 7 (20 °C), 6 (25 °C), 8 (30 °C), and 5 (ALL). Finally, five machine learning models (RF, SVR-RBF, SVR-POLY, XGBR, Lasso) were employed for regression analysis. The optimal hyperparameters were obtained using seven-fold cross-validation (see Table 2). The comparison between predicted and actual values on the test set is illustrated in Figure 2. In this study, models with R2 > 0.8 were considered suitable.

In the 15 °C group, all machine learning algorithms performed well, with SVR-RBF and XGBR exhibiting the best performance: SVR-RBF had the optimal R2 score (0.9154), MSE (32.1844), and RMSE (5.6731), while XGBR had the minimum MAE (3.9156). In the 20 °C group, RF, SVR-RBF, and XGBR performed well. The RF model had the best R2 score (0.8694), with optimal MSE (49.1035) and RMSE (7.0074). Like the 15 °C group, XGBR had the minimum MAE (4.5486). In the 25 °C group, SVR-RBF demonstrated the best performance with an R2 score of 0.8046, MSE of 65.5165, RMSE of 8.0942, and MAE of 6.0240. In the 30 °C group, all machine learning algorithms performed well, with XGBR showing the best performance. XGBR had an R2 score of 0.9460, MSE of 32.6807, RMSE of 5.7167, and MAE of 3.9733. When combining all temperature data, RF was the best-performing model (R2 score: 0.7583, MSE: 118.1160, RMSE: 10.8681, MAE: 7.0315). XGBR’s model was slightly inferior to RF.

## 4. Discussion

**Figure 3:**
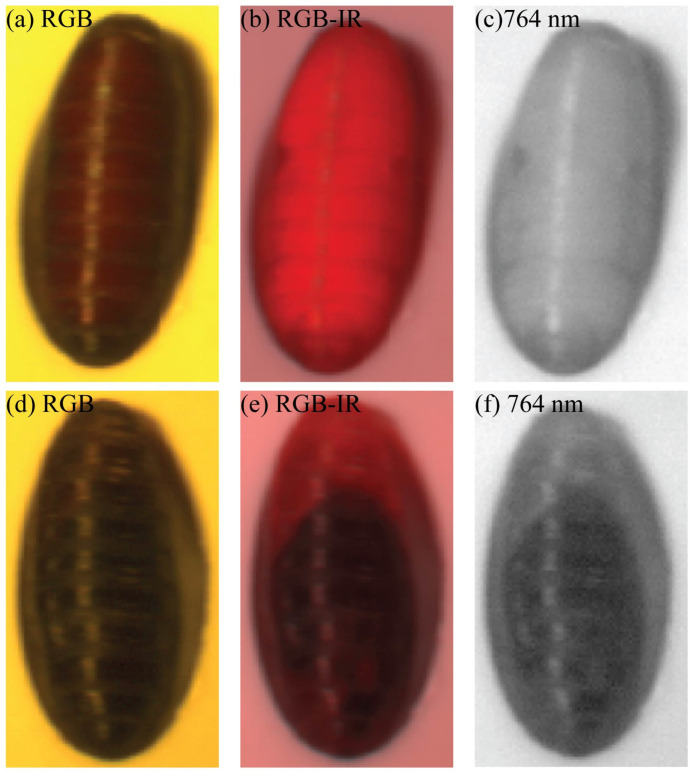
Spectral images of pupa. (**a**–**c**) Pupa at day 8 under 15 °C; (**d**–**f**) pupa at day 15 under 15 °C. RGB: Spectral images at RGB color pattern (Red, Green, Blue); RGB-IR: Spectral images at RGB-infrared; 764 nm: Spectral images at 764 nm.

This study elucidated the potential of HSI technology as a tool for estimating the age of blow fly pupa developing under various temperature conditions. Over time, changes in the physical and chemical structure of the cuticular layer of the pupa, along with corresponding alterations in reflectance, constitute a theoretical foundation for utilizing HSI in age determination during the pupa stage [28]. The pupa exoskeleton, derived from the cuticularization of the larval epidermis, is intricately linked to complex internal physiological processes crucial for water regulation, temperature modulation, and defense against pathogens. Prior research has demonstrated the variation of cuticular hydrocarbons in *Sarcophaga peregrina* over time [21]. Our result is similar to that which Voss et al. reported [28], indicating significant differences in reflectance curves among pupa of different ages and species. Analysis of average reflectance spectra of the pupa stage at various temperatures for *Calliphora vicina* revealed noticeable fluctuations in the average reflectance curve, closely associated with pupa age, though this variation is subtle [28]. Overall, there is a discernible decreasing trend in reflectance during the pupa stage, potentially linked to the gradual darkening of pupa color. In addition to the influence of the pupa exoskeleton on spectral reflectance, internal morphological changes within the pupa may contribute to variations in the average reflectance curve, as suggested in previous studies [28,34]. In our finding, HSI images, particularly in the 764 nm wavelength range, could capture partial morphological changes within the pupa (see Figure 3). Tao et al. reported a similar result, as they found that single-band images of silkworm pupa could observe the textural information of gonads at 500 nm [34]. Given the lack of comprehensive research in this domain, further exploration is warranted to ascertain the reasons behind these observations.

In our spectral data, across all temperatures, the pupa exhibited the highest average reflectance on the first day, likely attributed to the initial absence of cuticular tanning, resulting in a yellow-white appearance distinct from the mid-to-late pupa stages. This aligns with findings from Voss et al. [28], who proposed that the lack of key morphological changes, such as wings and legs, on the first day contributes to this phenomenon. Furthermore, we observed non-uniform declines in spectral average reflectance at the same temperature. For example, at 15 °C (chosen for its slower transitions), in the early stages, such as the first day, the average spectral reflectance was highest, gradually decreasing with pupa development. However, between days 2–12, while a decreasing trend persisted, daily reductions were not substantial. By day 13, there was a noticeable acceleration in the decline of average reflectance. Similar trends were evident at 20 °C and 25 °C, suggesting a collaborative effect of pupa cuticle color and internal morphology on this decline.

In this study, ADD served as a temporal scale for predicting development time, enabling the comparison of developmental data across different temperatures. ADD is widely utilized in insect developmental time estimation [17]. Given the high-dimensional nature of pupa spectral data involving 128 spectral bands within the 377–1035 nm range, traditional multivariate statistical analysis methods were inadequate for constructing suitable regression models. Consequently, machine learning methods were employed for model development. Our results indicated that RF, SVR-RBF, and XGBR models better use hyperspectral data in developmental time estimation. Among these, the XGBR model consistently exhibited the most minor errors, ranging between 3.9156 and 7.3951 (MAE). However, models such as SVR-POLY and Lasso failed to meet the requirements for estimation. Notably, the efficacy of different models varied, emphasizing the absence of a universally optimal machine-learning algorithm. While the field of entomology currently does not employ his in conjunction with machine learning for developmental time inference, it is noteworthy that scholars in diverse fields are actively investigating suitable regression models for hyperspectral data analysis [35]. For predicting winter wheat yields, employing hyperspectral sensitive bands as features and using Artificial Neural Network (ANN), Gaussian Process Regression (GPR), Multivariate Linear Regression (MLR), RF, Ridge Regression (RR), and SVM as base learners, with MLR as the meta-learner, an ensemble learning model yielded higher accuracy than individual algorithms [36]. Compared to PLS, SVM exhibited higher precision in estimating nitrogen content, crude protein content, and dry matter mass for leguminous crops and forage grasses [37]. These variations in optimal models underscore the importance of selecting appropriate models for pupa developmental time estimation.

This study represents the first application of machine learning algorithms and hyperspectral data for inferring the developmental stage of insect pupa. The results suggested thhisHSI data can be employed to estimate the developmental time of *Ch. megacephala* pupa. However, the sample size in this study requires further enhancement, and utilizing accumulated degree hours (ADH) as a temporal scale may enhance predictive accuracy because using ADD in the group of all temperatures in this study does not predict better than in individual temperature groups. Additionally, spectral scans of different parts of the pupa may yield variations, necessitating future research to focus on spectral changes in different pupa regions, offering a potential avenue for improving model accuracy. Furthermore, developing any predictive model necessitates validation in forensic practices under field conditions and relevant error rates. Blind testing in real-world scenarios and case studies is imperative to comprehensively establish the credibility of this technology in providing expert testimony in courtroom litigation. Portable hyperspectral detection instruments can now be easily taken to the crime scene for spectral detection of the pupa after adding machine learning models to the instrument to complete rapid non-destructive detection of the development time of the pupa, aiding TOC estimation.

## 5. Conclusions

This study reveals the potential of HSI in blow fly pupa age estimation. Machine learning models, notably RF, SVR with RBF kernel, and XGBR, could show promise in accurate developmental time estimation using accumulated degree days after data dimensionality reduction through PCA. However, the study acknowledges the need for a larger sample size, consideration of accumulated degree hours, and exploration of spectral changes in different pupa regions. This successful proof-of-concept study identifies the value of further refinement of the technique in forensic applications involving entomological specimens and identifies the considerable potential of HSI in forensic practice.

## Figures and Tables

**Table 1:** Data set.

Temperature	Data Set	Revised Data Set *	Training Set	Testing Set
15 °C	449	423	338	85
20 °C	241	220	176	44
25 °C	150	143	114	29
30 °C	150	149	119	30
All	990	935	748	187

* The revised data were obtained by removing outliers (95% confidence level) using the T^2^ Hotteling test.

**Table 2:** Evaluation metrics and hyperparameters of different models based on the testing set under different temperatures.

Temperature	PCs *	Evaluation Metrics					Hyperparameter
		ML Model	R2_Score	MSE	RMSE	MAE	
15 °C	6	RF	0.8777	46.5585	6.8234	4.7646	max_features = 4, n_estimators = 21
		SVR-RBF	0.9154	32.1844	5.6731	4.2668	C = 10000, gamma = 0.01, kernal = ‘rbf’
		SVR-POLY	0.8304	64.5724	8.0357	5.9882	C = 1000, degree = 1, kernal = ‘poly’
		XGBR	0.9069	35.4416	5.9533	3.9156	max_depth = 7, n_estimators = 1000
		Lasso	0.8265	66.0429	8.1267	6.2645	alpha = 0.5995, fit_intercept = Ture, max_iter = 1000
20 °C	7	RF	0.8694	49.1035	7.0074	4.7850	max_features = 6, n_estimators = 41
		SVR-RBF	0.8459	57.9510	7.6126	5.4913	C = 100, gamma = 0.01, kernal = ‘rbf’
		SVR-POLY	0.6432	134.1670	11.5830	8.9330	C = 1000, degree = 2, kernal = ‘poly’
		XGBR	0.8629	51.5522	7.1800	4.5486	max_depth = 7, n_estimators = 1000
		Lasso	0.7793	82.9672	9.1086	6.8378	alpha = 0.0215, fit_intercept = Ture, max_iter = 1000
25 °C	6	RF	0.7855	71.9127	8.4801	6.4624	max_features = 4, n_estimators = 51
		SVR-RBF	0.8046	65.5165	8.0942	6.0240	C = 10000, gamma = 0.001, kernal = ‘rbf’
		SVR-POLY	0.7302	90.4554	9.5108	7.1135	C = 100, degree = 1, kernal = ‘poly’
		XGBR	0.6607	113.7281	10.6643	7.1470	max_depth = 8, n_estimators = 100
		Lasso	0.7347	88.9378	9.4307	7.1464	alpha = 0.7743, fit_intercept = Ture, max_iter = 1000
30 °C	8	RF	0.9376	37.7242	6.1420	4.7838	max_features = 6, n_estimators = 51
		SVR-RBF	0.9359	38.7600	6.2258	5.1263	C = 1000, gamma = 0.001, kernal = ‘rbf’
		SVR-POLY	0.8988	61.1887	7.8223	6.7235	C = 100, degree = 1, kernal = ‘poly’
		XGBR	0.9460	32.6807	5.7167	3.9733	max_depth = 7, n_estimators = 1000
		Lasso	0.8918	65.4251	8.0886	6.7544	alpha = 0.2783, fit_intercept = Ture, max_iter = 1000
ALL	5	RF	0.7583	118.1160	10.8681	7.0315	max_features = 3, n_estimators = 91
		SVR-RBF	0.6041	193.4511	13.9087	7.6207	C = 10000, gamma = 0.01, kernal = ‘rbf’
		SVR-POLY	0.6111	190.0203	13.7848	10.6602	C = 100, degree = 1, kernal = ‘poly’
		XGBR	0.7449	124.6266	11.1636	7.3951	max_depth = 7, n_estimators = 100
		Lasso	0.6016	194.6762	13.9526	10.7460	alpha = 1, fit_intercept = Ture, max_iter = 1000

* The number of principal components (PCs) used in the model with the cumulative explanatory rate was >99%.

## Data Availability

The data and code presented in this study are contained in the manuscript and available on request from the corresponding author; therefore, they are not filed in a public repository.
